# Pneumomediastinum in a child with severe COVID-19

**DOI:** 10.1259/bjrcr.20200062

**Published:** 2020-11-26

**Authors:** Anne G Carroll, Nuala Quinn, Carl Byrne, Ghadir Kassab, Siobhan Whelan, Gabrielle C Colleran

**Affiliations:** 1 Department of Radiology, Children’s Health Ireland at Temple Street, Dublin, Ireland; 2 Emergency Department Research Group, Murdoch Children’s Research Institute, Melbourne, Australia; 3 Department of Paediatric Emergency Medicine, Children’s Health Ireland at Temple Street, Dublin, Ireland; 4 Department of Intensive Care Medicine, Children’s Health Ireland at Temple Street, Dublin, Ireland

## Abstract

The current global pandemic of the novel coronavirus SARS-CoV2 is a threat to the health and lives of millions of people worldwide. The latest statistics from the World Health Organisation show that there have been 6,515,796 confirmed cases worldwide with 387,298 confirmed deaths (last update 5 June 2020, 10:41 CEST). The majority of critically unwell patients with SARS-CoV2 are adults and the radiological findings associated with them are consistent throughout the literature. However, the reported paediatric cases are few, and as such, there is a limited body of evidence available. More international data is needed, not only on the clinical presentation, but also the radiological findings, so that health-care providers are better able to understand and diagnose this pandemic disease. We describe a case of a previously healthy 9-year-old female who presented to the Emergency Department with symptoms suggestive of raised intracranial pressure. Her CT revealed a medulloblastoma and post-operatively she tested positive for SARS-CoV2. She had a rapid deterioration in her clinical condition and required admission to the intensive care unit (ICU). We provide the supporting radiology along her clinical course in order to demonstrate important insights into this disease in children, including the unusual pnemomediastinum complications which occurred as part of her clinical course. This case is the first reported of its kind.

## Introduction

The impact of the current global pandemic of the novel coronavirus SARS-CoV2 has been unprecidented in modern history. It has placed a huge diagnostic and management burden on hospitals worldwide. The latest statistics from the World Health Organisation show that there have been 6,515,796 confirmed cases worldwide with 387,298 confirmed deaths (last update 5 June 2020, 10:41 CEST). The literature has been rapidly expanding. There is a growing body of evidence around the COVID-19 associated radiological findings. We know that adults are more likely than children to become critically unwell. Affected adults who develop acute respiratory distress syndrome (ARDS) need aggressive ventilatory support, develop multiorgan failure and may remain in intensive care units for weeks. Many health-care systems have a shortage of intensive care unit (ICU) and ventilator capacity during this pandemic. In contrast, what we know about SARS-CoV2 in children is limited. There is a paucity of paediatric evidence in the published literature and more case reports are needed. We describe a case of a 9-year-old female with a newly diagnosed medulloblastoma who tested positive for SARS-CoV2 post-operatively at the time of an acute respiratory deterioration. We provide the accompanying radiology along her clinical course.

## Case presentation

Our patient is a previously healthy 9-year-old female who presented to her local ED with sudden onset headache and drowsiness and a reduced Glasgow Coma Scale (GCS 12–14). A CT scan of her brain was performed which showed a large hyperdense midline lesion centred on the fourth ventricle with associated obstructive hydrocephalus and transependymal oedema. She had no past medical history, was afebrile and had no respiratory symptoms. Her family had no known sick contacts. Shortly after CT, she deteriorated and became unconscious requiring endotracheal intubation by rapid sequence induction. She was immediately transferred to our tertiary paediatric neurosurgical centre with a new diagnosis of a posterior fossa tumour.

**Figure 1.**
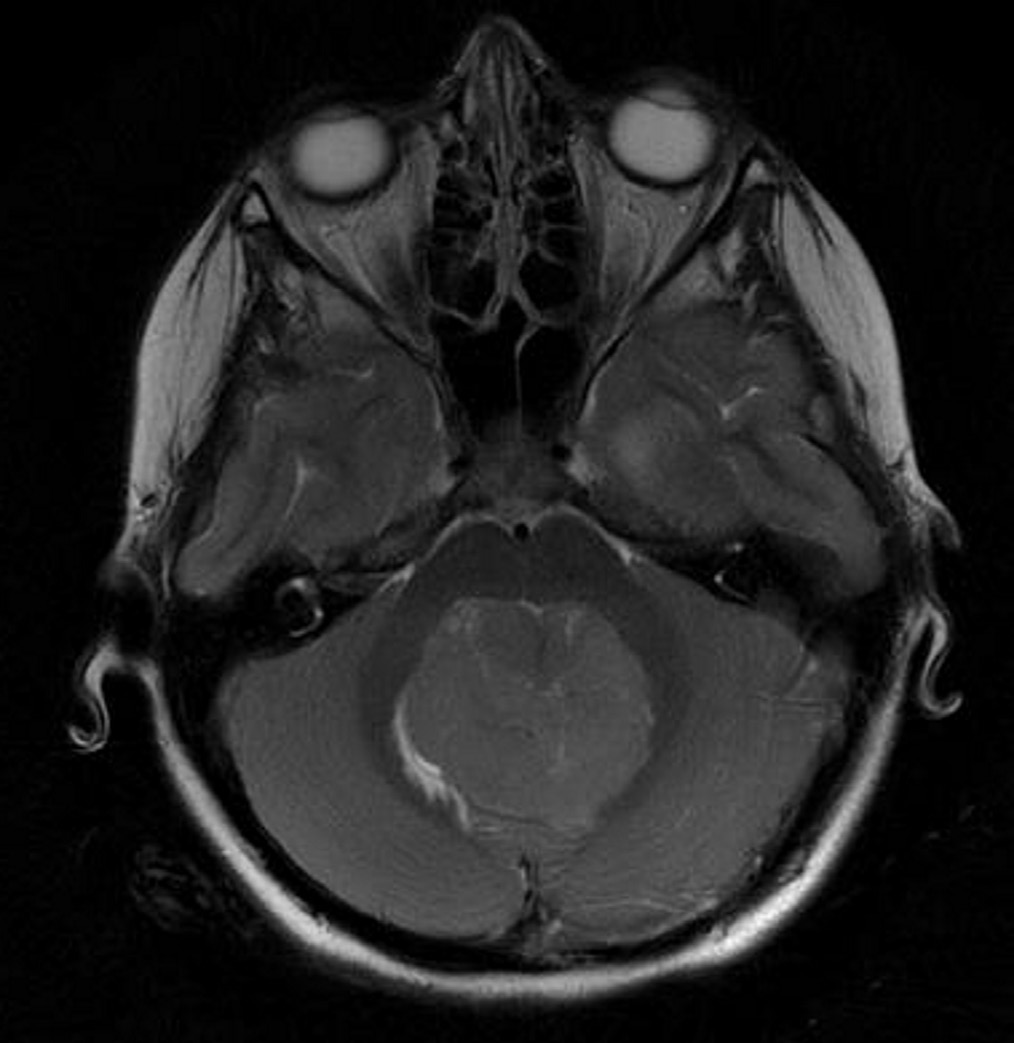
Axial *T_2_* weighted image of the posterior fossa which demonstrates a mildly T2 hyperintense midline mass in the fourth ventricle, subsequently confirmed on histology to be a medulloblastoma.

Shortly after her arrival to our hospital, Children's Health Ireland at Temple Street, she had an external ventricular drain (EVD) inserted in theatre and was commenced on intravenous dexamethasone 2 mg bd (weight 26 kg). She underwent a MR scan of her brain, which demonstrated an enhancing, T2 hyperintense midline mass centred on the fourth ventricle (Figure 1) demonstrating reduced diffusivity. This was suspicious for a medulloblastoma. The initial post-EVD insertion course was uneventful and she was extubated successfully.

2 days later (Day 3) she underwent a suboccipital craniectomy and subtotal resection of tumour. Her pre-operative chest X-ray (CXR) was normal. She had minimal blood loss during the surgery and post-operative MRI revealed no evidence of vascular insult. Given the significant cerebral oedema intraoperatively, it was decided to delay dural and skull closure. Histological analysis of the resected tumour revealed a malignant small round blue cell tumour. Immunohistochemistry demonstrated a synaptophysin positive profile. Histological and radiology findings conclude that the lesion was a non-WMT non-SHH medulloblastoma.

**Figure 2.**
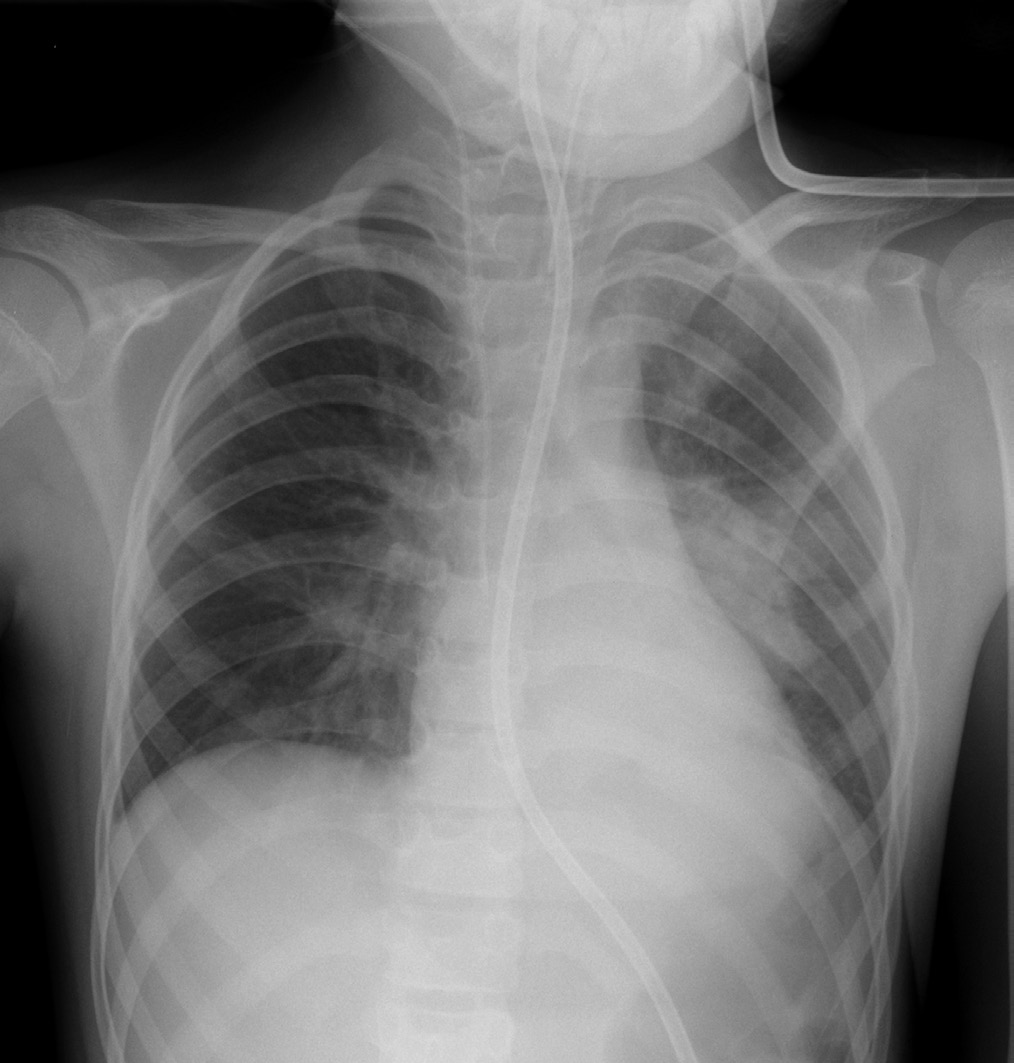
Day 2 post-operative. Allowing for some rotation of the patient, CXR shows new airspace opacification in the left mid zone and left lower lobe, consistent with pneumonia and atelectasis. CXR, chest X-ray.

Her post-op extubation was deferred due to copious thick secretions and high ventilatory requirements during her operation. A bronchoscopy demonstrated thick secretions bilaterally, which were suctioned leading to a clinical improvement. Endotracheal aspirates showed no bacterial growth. CXR showed pneumonia and atelectasis in the left mid zone and left lower lobe (Figure 2). She was extubated the following day. At that time, her respiratory examination demonstrated mild reduced air entry to the left lower lobe and mild bilateral wheeze. A SARS-CoV2 swab was sent. She remained afebrile. At this time, her full blood count (FBC) and C-reactive protein (CRP) were within normal limits.

**Figure 3.**
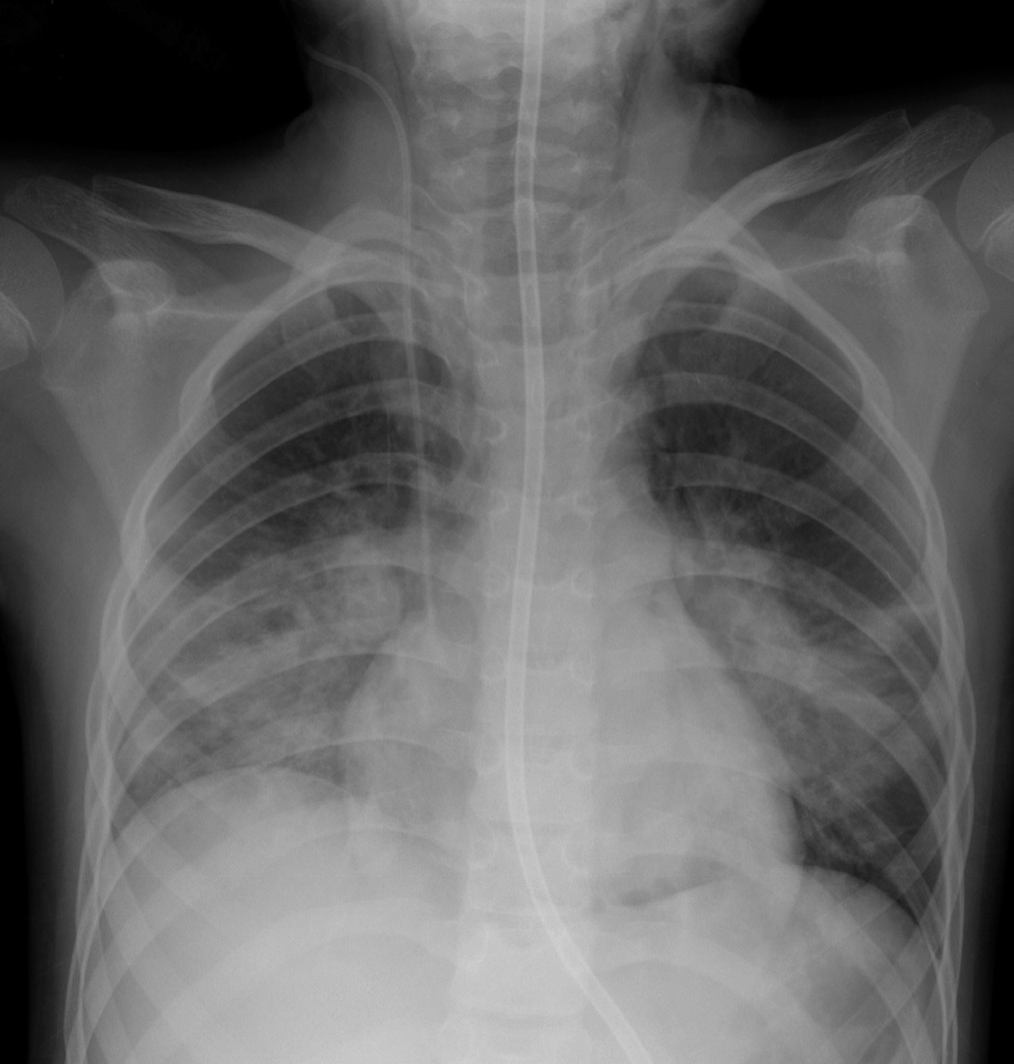
Day 5 post-operative. CXR shows interval progression of confluent airspace opacities in both mid and lower zones. The patient developed pneumomediastinum at this point. Note subcutaneous emphysema tracking along the left side-of the neck. CXR, chest X-ray.

The swab was reported positive for SARS-CoV2 the next day. Two days after (Day 5 post-operative), there was a marked deterioration in her clinical status. She became tachypnoeic and had an increase in the severity and production of her cough. She had frequent episodes of hypoxia (SpO_2_ 76%) despite being on high flow nasal oxygen. She became neutrophilic during this initial deterioration. Neutrophils were elevated at 13.34. Her lactate dehydrogenase (LDH) and coagulation profile also were significantly deranged with her LDH, prothrombin time and activated partial thromboplastin time elevated at 1028 U l^−1^, 14.0 s and 22.0 s respectively. Her FiO_2_ was increased to 35% and nebulised salbutamol was administered. Despite these interventions, her oxygen requirements increased to FiO_2_ 50%. Large volumes of thick secretions were noted. CXR showed progression of confluent airspace opacities in both mid and lower zones and also new pneumomediastinum (Figure 3). She was admitted to the ICU. Despite the large volumes of secretions, cultures from samples taken during bronchoalveolar lavage (BAL) did not demonstrate any clear bacterial growth, with commensal bacteria being identified at counts < 10,000 μ/l.

**Figure 4.**
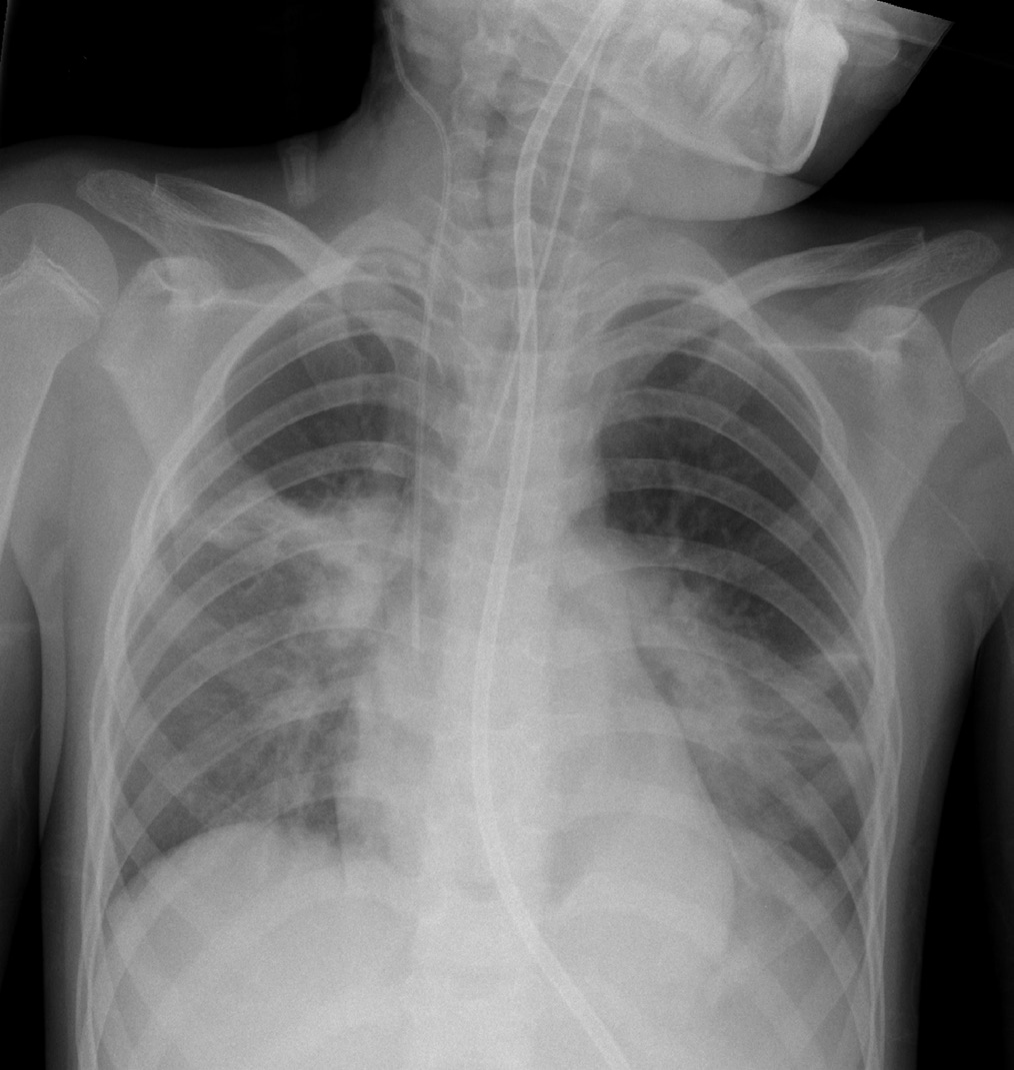
Day 7 post-operative. CXR shows improved aeration of the right lower zone with progressive airspace opacity in the right mid zone. Pneumomediastinum is still present. CXR, chest X-ray.

Over the subsequent days she became more hypoxic, with frequent episodes of desaturation to 80%. She was re-intubated on Day 7 post-operatively. There was a significant increase in thick purulent respiratory secretions which were removed under bronchoscopy. CXR showed development of new airspace opacity in the right mid zone (Figure 4).

**Figure 5.**
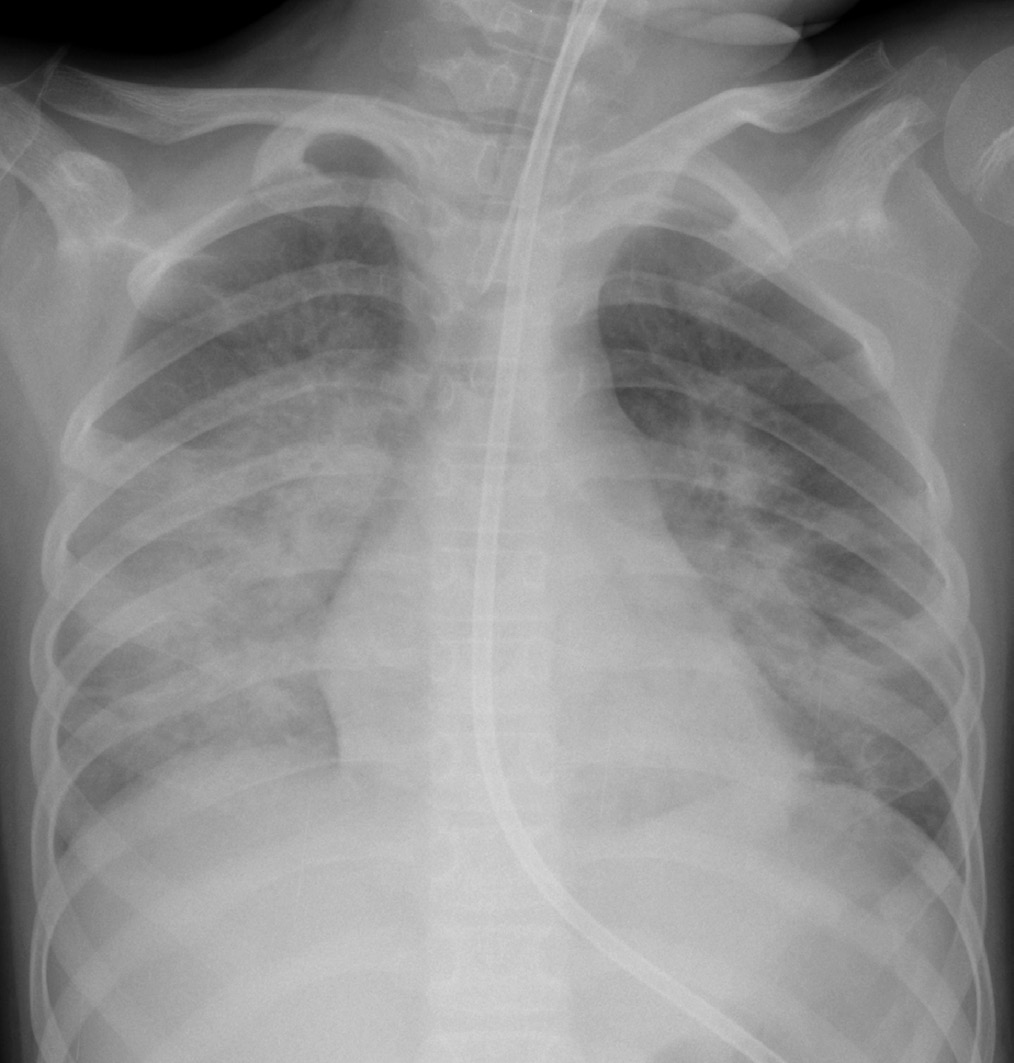
Day 13 post-operative. CXR shows progression of right mid and lower zone airspace opacities. The left lung appearance is stable. CXR, chest X-ray.

**Figure 6.**
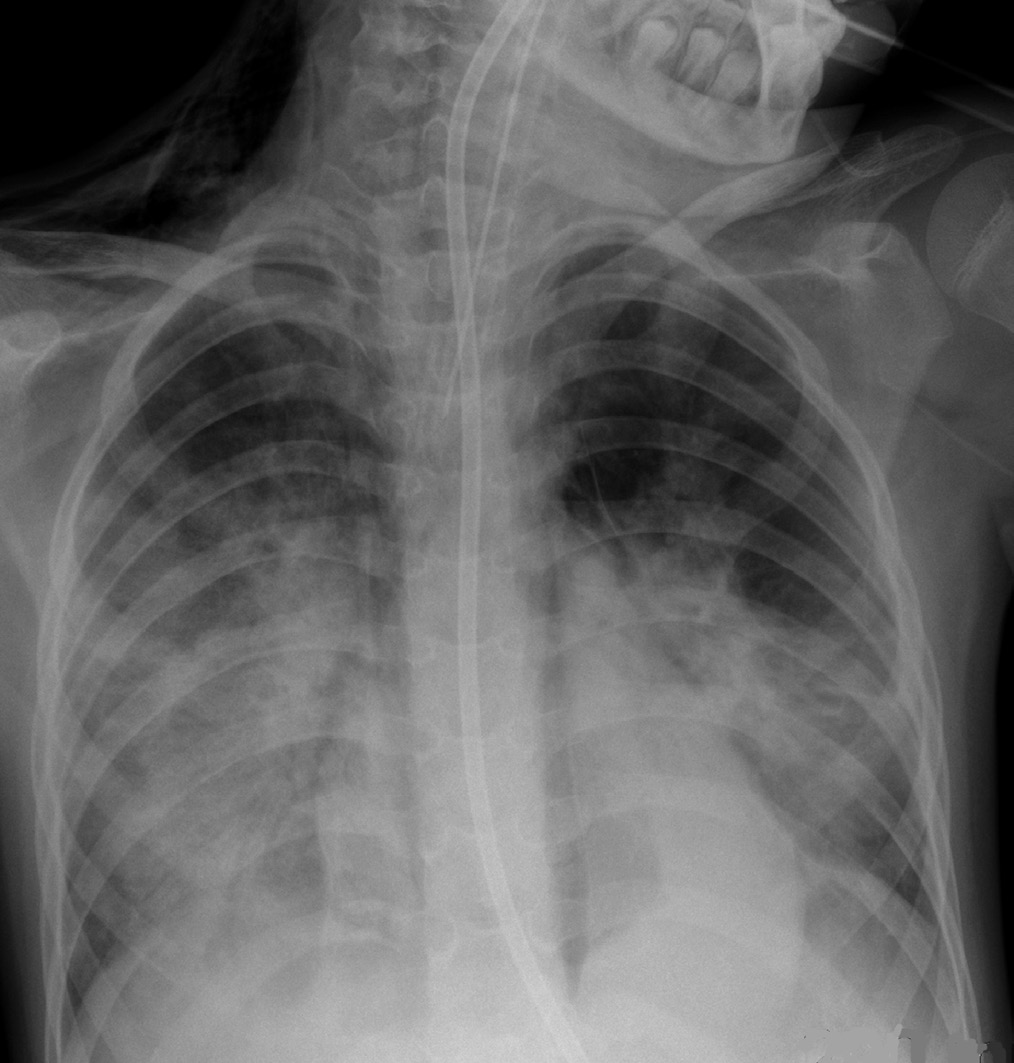
Day 16 post-operative. CXR shows stable air space opacification bilaterally. The child has developed recurrent pneumomediastinum, while intubated. CXR, chest X-ray.

The patient remained ventilated for 25 days. She required increasing ventilatory support and was having episodes of hypoxia, with reduction in SpO_2_ to 89%. Her radiography was consistent with this deterioration, with progression of right mid- and lower zone airspace opacities (Figure 5) and development of recurrent peumomediastinum (Figure 6). Clinical examination at this time revealed mildly reduced air entry bilaterally with some transmitted sounds. Her blood profile was now abnormal with an elevated white cell count (20.3 × 10^9^/l) and C-reactive protein 203 mg l^−1^.

**Figure 7.**
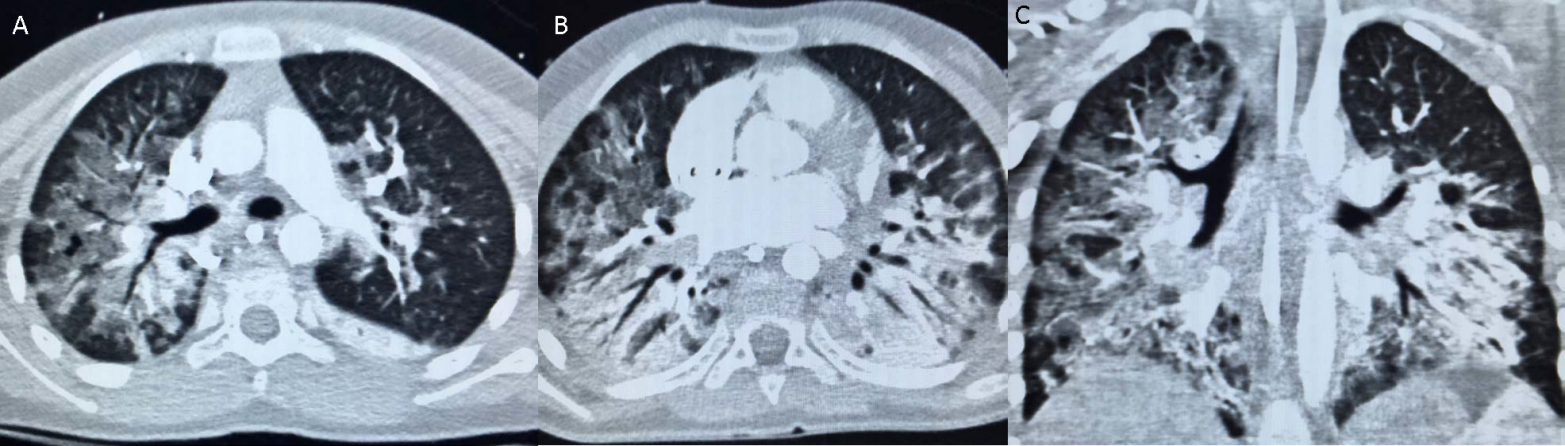
A–C: Day 26 post-operative. Contrast-enhanced helical CT Thorax, KV 100, MA148, 2mm slice thickness performed under general anaesthesia. Paediatric dose reduction as per patient weight.

She had a contrast-enhanced CT thorax which demonstrated bilateral parahilar infiltrates and bilateral dependant consolidation in keeping with ARDS. There was diffuse ground glass opacity in the right lung with relative peripheral sparing, but no significant ground glass opacity in the left lung (Figure 7).

There followed a slow improvement. She responded well to prone positioning. Her ventilator settings were reduced and there was significant reduction in endotracheal secretions. Her inflammatory markers normalised. Her clinical condition improved and she was extubated 4 days later (post-operative Day 29).

**Figure 8.**
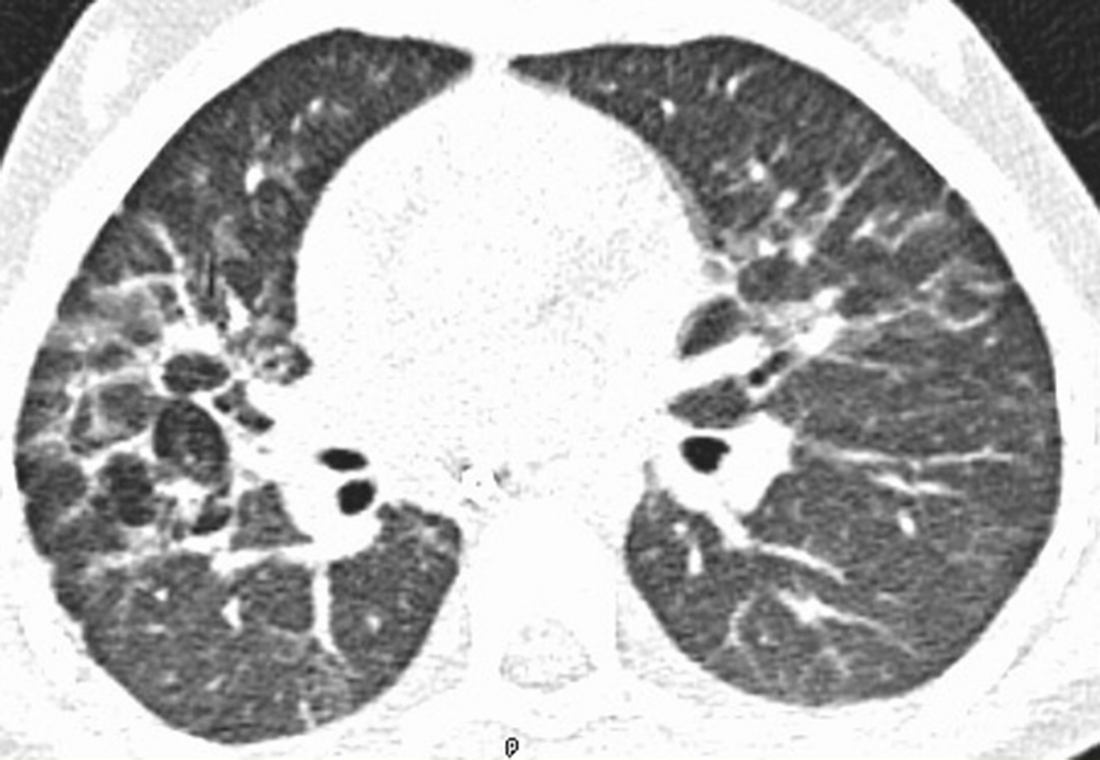
Follow-up high resolution CT Thorax obtained 6 weeks after CT. KV 100 MA 60, paediatric dose reduction. Axial image on lung windows demonstrates bilateral linear opacities in a peribronchovascular distribution, right side worse than left. The appearances are suggestive of persistent inflammatory changes and possible early fibrosis.

She made a slow clinical recovery over the subsequent number of weeks. A follow-up high resolution CT Thorax performed 6 weeks after the initial CT Thorax showed bilateral peribronchovascular linear densities, suggestive of persistent inflammatory changes and possible early fibrosis (Figure 8). She will continue to have respiratory follow-up. She went on to commence chemotherapy and radiotherapy for further treatement of her medulloblastoma.

## Discussion

To date, there has been limited evidence published regarding the presentation, clinical course and imaging findings in children infected with COVID-19. Many children appear to be asymptomatic. Some present with fever, cough, sore throat, respiratory distress or gastrointestinal symptoms. Severe COVID-19 infection requiring ICU admission appears to be much less common in children than in adults.^1–6^ Imaging findings in children range from normal CXR and CT thorax, to findings similar to adults, including ground glass opacities, consolidation and appearances typical of ARDS.^3,7,8^ The incidence of peribronchial thickening and parahilar distribution of infiltrates appears to be more common in children compared with adults, with the typical subpleural ground glass opacities seen in adults less prevalent in children.^8^

Our patient had no respiratory signs or symptoms when she presented to the ED with symptoms of her brain tumour. Her initial CXR showed normal appearance of both lungs. Her respiratory symptoms only started in the early post-operative period after neurosurgical resection of a medulloblastoma. Serial chest radiography demonstrated worsening consolidation, initially in a parahilar distribution, with a changing “fleeting” distribution of consolidation on serial radiographs, as well as the development of pneumomediastinum. She progressively deteriorated, requiring mechanical ventilation. Her CT thorax subsequently demonstrated ground-glass opacities and dependant consolidation consistent with ARDS, as is commonly seen in severe COVID-19.

It is possible that this patient’s COVID-19 infection may have been more severe after having undergone major surgery, as has been reported in some small case series around the world.^9–11^ She received 14 days of steroid treatment for raised intracranial pressure pre- and post-resection of a medulloblastoma. There is emerging evidence of the beneficial effect of steroid treatment on patients with COVID-19. The possiblity of a concurrent bacterial infection was a concern following the patients initial deterioration post-operatively. BAL cultures demonstrated no clear bacterial co-infection. with growth of commensals at counts of <10,000 μ/l from her initial BAL. Three further BAL cultures demonstrated no bacterial growth over the subsequent fortnight despite the prevelance of copious secretions during the procedure.

An unusual aspect of this case is the occurrence of pneumomediastinum on two separate occasions; initially when the patient first clinically deteriorated prior to intubation and transfer to ICU and the second episode occurred later in the clinical course when the patient was ventilated in the ICU. Pneumothorax or pneumomediastinum have been rarely reported in Covid-19 patients and, to our knowledge, only in adults.^5,12^ Interestingly, Zhou et al described a case report of a 38-year-old male in Wuhan who was on steroids at the time of pneumomediastinum, although he was also ventilated at the time.^12^ This case is similar to another case report of a 38-year-old male reported by Sun et al who presented on the same day to the same hospital in Wuhan^13^; given the similarities in age and location and temporal association, it is thought possible that these publications may represent the same patient. Chen et al report one case of a patient with pneumothorax also in Wuhan in January 2020, but no further details are provided.^5^

We hypothesise that alveolar damage secondary to coronavirus infection may be the aetiology behind the possible association with pneumothorax and pneumomediastinum in severe COVID-19. However, larger case series would be necessary before association could be confirmed and there is currently insufficient evidence to demonstrate causation. We feel that it is unlikely that pneumomediastinum was due to mechanical ventilation or otherwise iatrogenic in this patient as the initial pneumomediastinum occurred during a period of self ventilation with no air leak evident on the preceding CXR (Figure 2) and no new central lines were inserted in that interval. Atelectasis is a common finding in post-operative paediatric patients, but the unusual distribution of airspace opacification and the presence of pneumomediastinum in this patient alerted the reporting radiologist to consider COVID-19, prior to the patient’s swab result being available. Given our experience in the setting of community transmission of COVID-19, we recommend that the presence of spontaneous pneumomediastinum should cause the clinician to consider the diagnosis.

## Conclusion

This case provides a detailed insight into the clinical presentation and radiological features of a child with severe COVID-19 infection, including an atypical presentation with pneumomediastinum during a period of self ventilation. Further research is needed to demonstrate association and possible causation.

## Learning points

- During the current pandemic, beware of possible COVID-19 infection in previously asymptomatic patients who suddenly clinically deteriorate in the post-operative period.
- COVID-19 infection may be more severe in children who become infected in the immediate pre-operative or perioperative period, as has been seen in adults.
- Air leak complications such as spontaneous pneumomediastinum may be associated with COVID-19 infection. Case reports are merely hypothesis generating. More research is needed to study this possible association.
